# Troy is expressed in human stomach mucosa and a novel putative prognostic marker of intestinal type gastric cancer

**DOI:** 10.18632/oncotarget.10672

**Published:** 2016-07-18

**Authors:** Franziska Wilhelm, Christine Böger, Sandra Krüger, Hans-Michael Behrens, Christoph Röcken

**Affiliations:** ^1^ Department of Pathology, Christian-Albrechts-University, Kiel, Germany

**Keywords:** Tnfrsf19 (Troy), Wnt signaling, stomach, gastric cancer, differentiation

## Abstract

Epithelial stem cells of gastrointestinal tissues are characterized and controlled by an active Wnt signaling. Recently, the Wnt target gene *Troy* has been proposed as a neoplastic marker in the murine intestine. In this study, we explored the putative tumor biological significance of Troy in humans by using immunohistochemistry (104 cases), quantitative RT-PCR (50 cases) and cell culture experiments (MKN45, MKN74). In the non-neoplastic gastric mucosa, Troy was expressed by Muc5AC-positive foveolar epithelium, parietal cells, chief cells and cells of the intestinal metaplasia. In gastric cancer, Troy was found in the desmoplastic stroma and tumor cells. The overall staining intensity of the tumor cells was lower compared with the adjacent non-neoplastic mucosa, Troy was found significantly more commonly in intestinal compared with diffuse type gastric cancer (p=0.001) and correlated inversely with tumor grade (p<0.001) and nodal spread (p=0.025). In the intestinal type, loss of Troy-expression was associated with a significantly worse overall survival (p=0.006). Subsequent cell culture experiments showed a Wnt dependent expression of Troy and a reduced colony formation ability of Troy-overexpressing MKN45-cells. Our results lead to the conjecture that Troy is also a negative regulator of WNT signaling in gastric cancer, which affects patient outcome.

## INTRODUCTION

Gastric cancer (GC) constitutes the third most common death-causing cancer worldwide and remains one of the most fatal diseases due to its late diagnosis resulting in limited therapeutic options [1–3]. Insights into the underlying regulation of gastric stem cells and their counterparts in cancer are crucially needed to improve the outcome of this highly heterogeneous disease. In general, stem cells of the gastrointestinal tract mainly depend on Wnt signaling and aberrant activation of this pathway has been linked to a significant proportion of gastric cancer cases [4, 5]. In mouse models, experimental Wnt signal deregulation (such as loss of APC function) readily leads to the appearance of adenomas [6]. Likewise, frame shift mutations within the Wnt signaling suppressors APC or Rnf43 as well as activating mutations of β-catenin have been identified as driver mutations in human GCs [7, 8].

Troy (syn. for Tnfrsf19), a member of the TNF-receptor superfamily, is one Wnt target gene frequently found within gastrointestinal LGR5^+^ stem cell expression profiles [9, 10]. This type I transmembrane receptor is located within the plasmamembrane but lacks the classical death domain of the Tnfr superfamily. Troy is widely expressed during embryonic development but becomes restricted mainly to the central nervous system and the developing hair follicle in the adult [11, 12]. Functionally, Troy is able to fulfill pleiotropic roles depending on the cellular context. In the hair follicle as well as tumor-associated microglia, Troy acts as homologue to Edar by controlling the NFkB pathway [13, 14]. In the central nervous system, Troy functionally replaces p75 to inhibit axon outgrowth via EGFR/RhoA signaling [15, 16] or activates Rac1 to facilitate cell migration in advanced glial tumors [13, 17]. Within gastrointestinal stem cells, Troy gained another important function by binding to LGR5. This was shown to inhibit intracellular Wnt signaling by influencing the Wnt co-receptor Lrp6 [9]. Thereby Troy expression establishes a negative feedback loop to control Wnt signal strength in order to maintain a coordinated stem cell function, playing an important role within the gastrointestinal tissue homeostasis.

Interestingly, *Troy* knockout mice are viable without obvious phenotype [14], suggesting that its loss is compensated by redundant functions. Nevertheless, dysregulation of Troy expression has been reported for several malignant cancer entities like melanomas [18], glioblastomas [17], and squamous cell carcinomas [19]. Within the murine intestine, Troy has also been established as a tumor marker of neoplastic tissues. However, these results could not be recapitulated in human colorectal cancer [9] or only a trend was stated [20]. For the stomach, only little data from mouse studies is available. Contrary to the intestine, Troy expression was mainly attributed to a subset of parietal and chief cells at the gland base, of which the differentiated Troy^+^ chief cells proved their capability to regenerate the whole epithelium in lineage tracing experiments, implying their ability to dedifferentiate and act as a reserve stem cell population [21].

We hypothesize that Troy is also a member of and regulated by the Wnt-signaling complex in the human stomach and hence might be involved in gastric cancer biology. In order to prove our hypothesis, we explored the expression pattern of Troy in human non-neoplastic and neoplastic stomach mucosa on transcriptional and translational level, correlated the expression with various clinico-pathological patient characteristics, including patient survival, and carried out cell culture experiments in transfected and un-transfected gastric cancer cell lines. Collectively our data provide evidence that Troy is down-regulated in gastric cancer, is an inverse prognosticator of poor patient outcome and is expressed in a WNT dependent manner.

## RESULTS

### Troy is expressed in the human gastric mucosa

**Figure 1:**
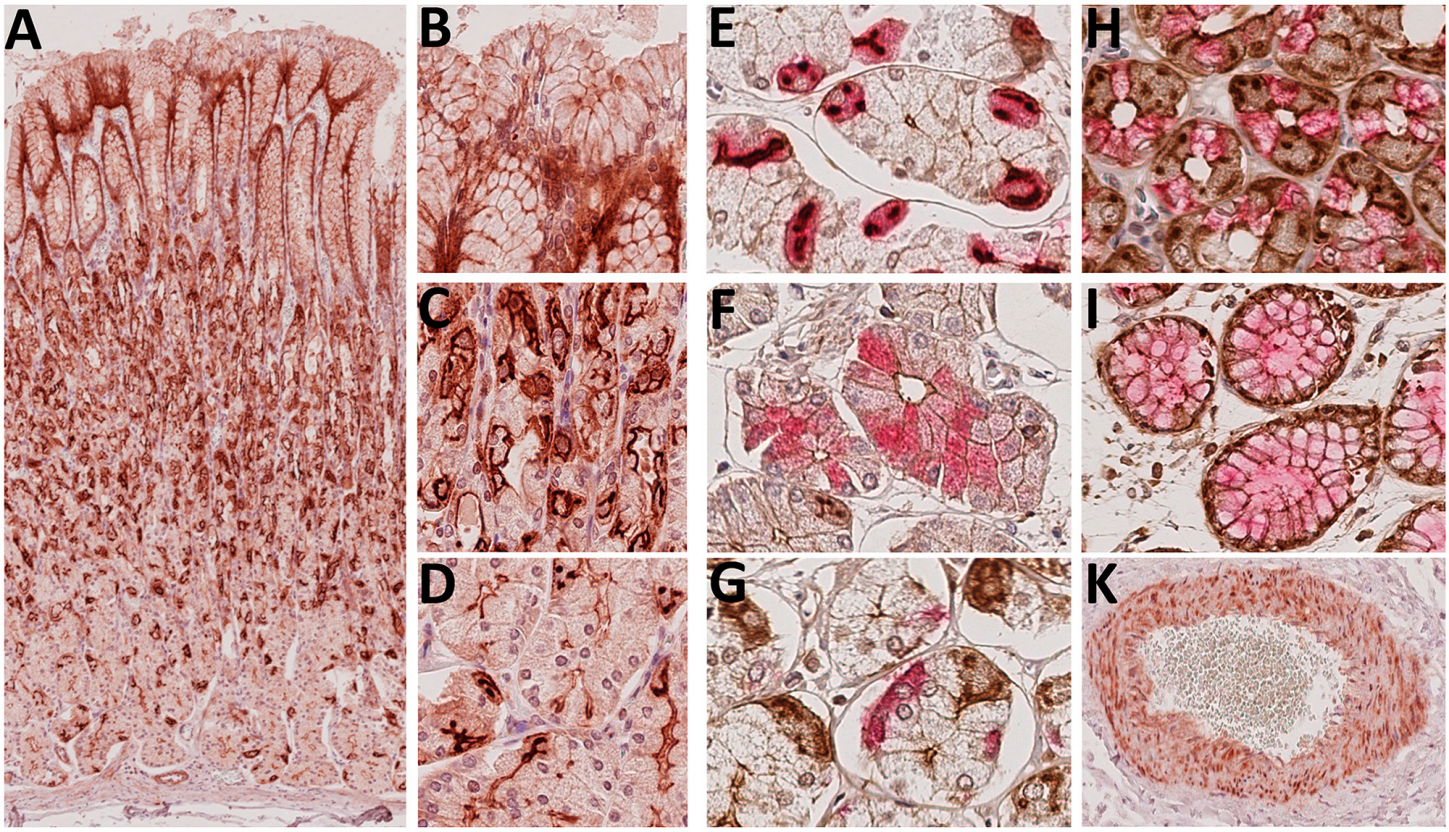
Immunohistochemical detection of Troy^+^ cells in the gastric corpus mucosa **A-D**. Whole section overview of the stomach epithelium. As expected, nuclei were immunonegative (original magnification A: 60x, inserts B-D: 250x). **E-I**. Identification of Troy^+^ cells (brown DAB staining) by double staining with lineage markers (VectorRed): gastric H^+^/K^+^-ATPase (E), pepsinogen C (F), chromogranin A (G), Muc6 (H) or Muc5 (I); original magnifications 400x. **K**. Troy^+^ myocytes allowed for internal staining control.

First we studied the histoanatomical expression of Troy in the healthy human stomach mucosa by immunohistochemical staining of five sleeve gastrectomy specimens. Unexpectedly, we found Troy widely expressed throughout the gastric epithelial layer (Figure 1A-1D). Troy was also found in myocytes, a phenomenon that resembled the expression profile of the Troy-eGFP-IRES-CreERT2 mouse model [21] and served as internal staining control (Figure 1K). By double staining techniques, intensively Troy expressing cells were identified as Muc5AC positive cells of the foveola (Figure 1I). In the isthmus and neck area, prominent Troy expression appeared in parietal cells (Figure 1E), where the protein was accumulated in the canaliculi of the secretory network. As the highest density of parietal cells was found in the corpus, Troy staining appeared most prominent in this region. Nevertheless, the functional localization of Troy is expected at the plasmamembrane, where it was found with an apical orientation mainly in chief cells (Figure 1F), whereas enteroendocrine cells (Figure 1G) and Muc6 positive cells (Figure 1H) did not express Troy. Taken together, the pronounced Troy immunostaining comprised an unexpected large proportion of differentiated epithelial cells of the human stomach tissue.

### The relevance of Troy expression in gastric cancer

**Figure 2:**
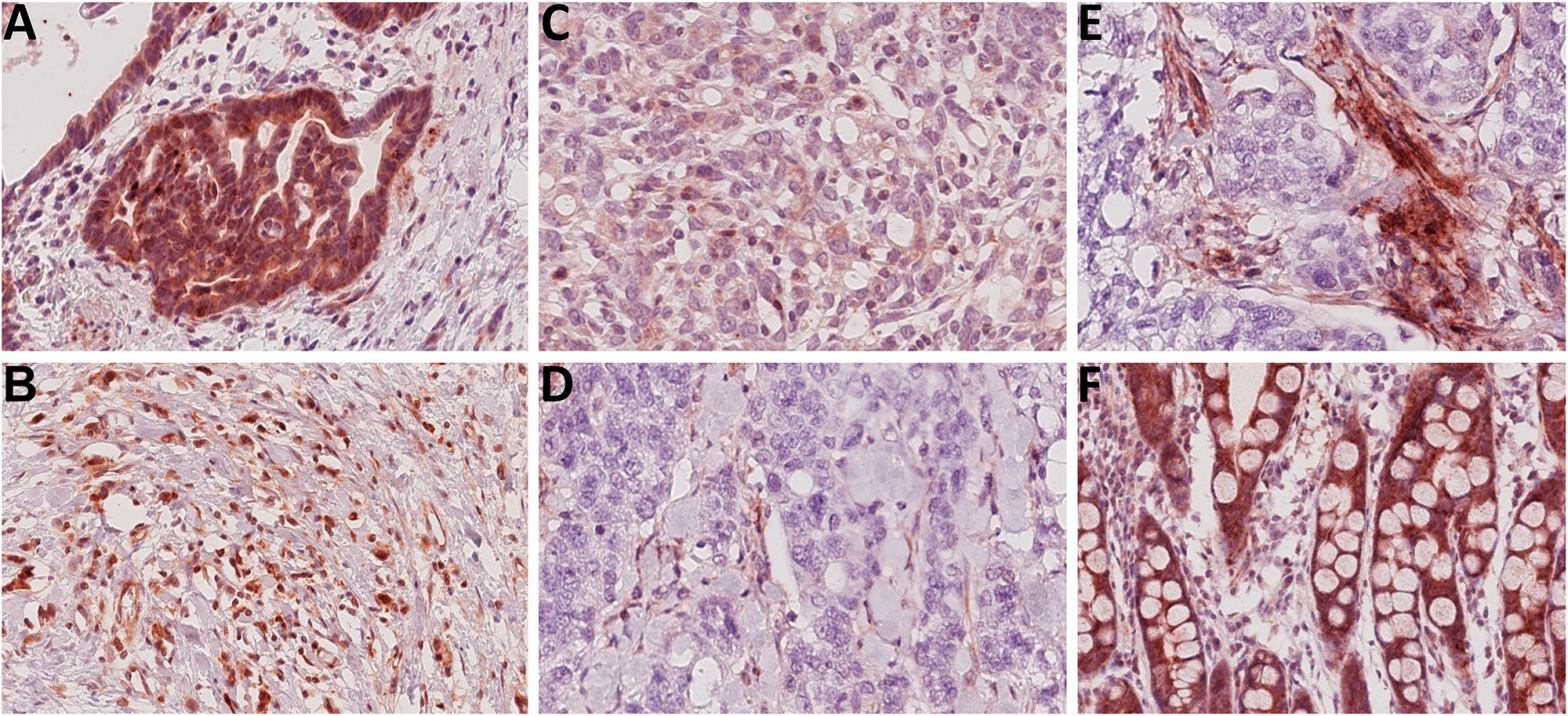
Troy expression within gastric cancer tissues **A-D**. Representative immunostainings of high (A+B), moderate (C) and low (D) Troy expression in tumor cells of intestinal (A+D) or diffuse type (B+C) gastric cancer. **E**. Analysis of Troy immunostaining of tumor cells was complicated by a noticeable expression within stromal components. **F**. Most intestinal metaplasia (17 of 19 cases) stained positive for Troy (all original magnifications 250x).

**Figure 3:**
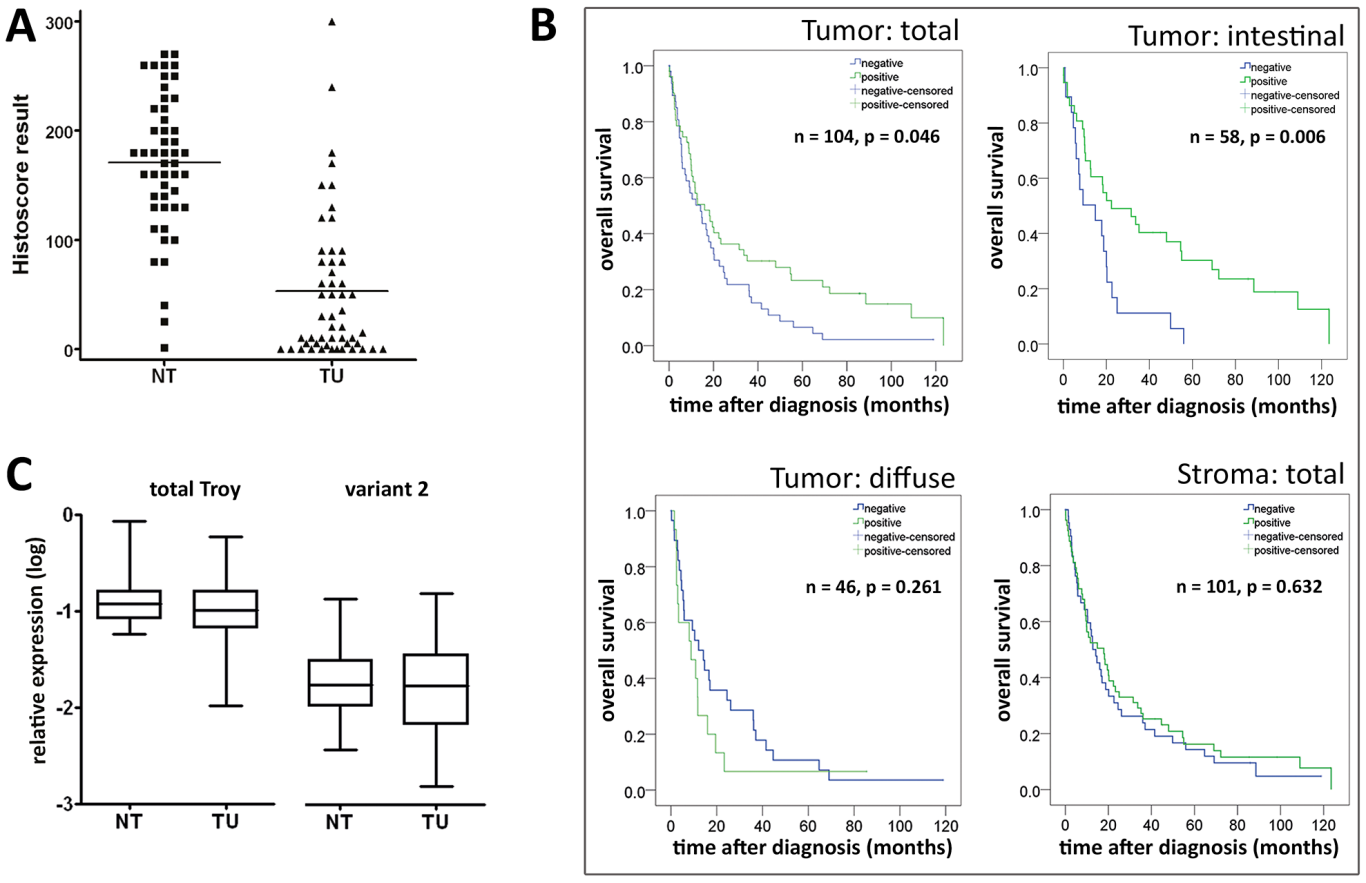
Detection of Troy on protein level is relevant for gastric cancer prognosis **A**. Distribution of the Histoscore results for non-neoplastic mucosal epithelium (NT) and corresponding cancer cells (TU; n=52). Compared with normal tissue, neoplastic cells showed a less pronounced Troy expression. For further analysis, the group was divided at the median (positive, when Histoscore ≥ 50, compared to low expressing or negative cases). **B**. Survival analysis for Troy-positive and Troy-negative patients using Kaplan-Meier plots. Troy expression proved especially relevant for prognosis of intestinal type GC, whereas stroma cell expression had no obvious predictive power. **C**. qRT-PCR analysis on tumor samples and matching non-neoplastic mucosa with primers detecting all isoforms of Troy (left) or specifically transcript variant 2 (right). Expression was normalized to *succinate dehydrogenase subunit A* (SDHA), *calpain 2* (CAPN2) and *cyclophilin C* (CYCC) as housekeeping genes and logarithmized (n=50).

Next we studied Troy expression in GC specimens obtained from 104 patients. Corresponding non-neoplastic gastric mucosa was available from 52 patients (see Table 1 for patient characteristics). Compared with parietal cells, tumor cell staining was less intense and mainly homogenously distributed throughout the cytoplasm (Figure 2A-2D), resulting in a significantly decreased Histoscore of the tumor cells compared with the non-neoplastic epithelium in 52 matched tissue samples (tumor vs. non-tumor)(Figure 3A).

**Table 1:** Correlation of Troy expression (divided into groups of positive and low/negative cells at the median Histoscore result) with clinico-pathological patient characteristics of the gastric cancer cohort

	Tumor cells			Stroma cells		
	total	Troy low/neg n (%)	Troy positive n (%)	total	Troy low/neg n (%)	Troy positive n (%)
**Number of patients**	104	51 (49.0)	53 (51.0)	101	47 (46.5)	54 (53.3)
**Age**			***p=0.555***			***p=0.843***
<68 years	47	25 (53.2)	22 (46.8)	46	22 (47.8)	24 (52.2)
≥68 years	57	26 (45.6)	31 (54.4)	55	25 (45.5)	30 (54.5)
**Gender**			***p=0.316***			***p=0.415***
female	41	23 (56.1)	18 (43.9)	40	21 (52.5)	19 (47.5)
male	63	28 (44.4)	35 (55.6)	61	26 (42.6)	35 (57.4)
**Lauren classification**			***p=0.001^#^***			***p=0.001^#^***
intestinal	58	20 (34.5)	38 (65.5)	58	18 (31.0)	40 (69.0)
diffuse	46	31 (67.4)	15 (32.6)	43	29 (67.4)	14 (32.6)
**T category**			***p=0.047^a^***			***p=0.168^a^***
pT1a/b	11	3 (27.3)	8 (72.7)	10	3 (30.0)	7 (70.0)
pT2	14	4 (28.6)	10 (71.4)	14	5 (35.7)	9 (64.3)
pT3	40	22 (55.0)	18 (45.0)	39	19 (48.7)	20 (51.3)
pT4a/b	39	22 (56.4)	17 (43.6)	38	20 (52.6)	18 (47.4)
**N category**			***p=0.025^a,#^***			***p=0.067^a^***
pN0	33	10 (30.3)	23 (69.7)	32	11 (34.4)	21 (65.6)
pN1	16	10 (62.5)	6 (37.5)	16	7 (43.8)	9 (56.2)
pN2	11	6 (54.5)	5 (45.5)	11	5 (45.5)	6 (54.5)
pN3a/b	43	25 (58.1)	18 (41.9)	41	23 (56.1)	18 (43.9)
**M category**			***p=0.434***			***p=0.603***
pM0	87	41 (47.1)	46 (52.9)	84	38 (45.2)	46 (54.8)
pM1	17	10 (58.8)	7 (41.2)	17	9 (52.9)	8 (47.1)
**UICC-Stage**			***p=0.026^a^***			***p=0.133^a^***
IA/B	16	4 (25.0)	12 (75.0)	15	4 (26.7)	11 (73.3)
IIA/B	25	11 (44.0)	14 (56.0)	25	11 (44.0)	14 (56.0)
IIIA/B/C	45	26 (57.8)	19 (42.2)	43	22 (51.2)	21 (48.8)
IV	17	10 (58.8)	7 (41.2)	17	9 (52.9)	8 (47.1)
**Grade**			***p=0.000^#^***			***p=0.045***
G1/G2	41	9 (22.0)	32 (78.0)	41	14 (34.1)	27 (65.9)
G3/G4	63	42 (66.7)	21 (33.3)	60	33 (55.0)	27 (45.0)
**R-status**			***p=0.695***			***p=1.000***
R0	95	47 (49.5)	48 (50.5)	93	43 (46.2)	50 (53.8)
R1/R2	7	4 (57.1)	3 (42.9)	6	3 (50)	3 (50)
**E-Cadherin**			***p=0.003^#^***			***p=0.080***
negative	67	41 (61.2)	26 (38.8)	66	35 (53.0)	31 (47.0
positive	32	9 (28.1)	23 (71.9)	31	10 (32.3)	21 (67.7)
**β-Catenin**			***p=0.003^#^***			***p=0.000^#^***
negative	49	32 (65.3)	17 (34.7)	47	33 (70.2)	14 (29.8)
positive	50	17 (34.0)	33 (66.0)	50	13 (26.0)	37 (74.0)

Data was analyzed by the 2-sided Fisher's Exact Test until not stated otherwise (a: tested with Kendall's Tau test; #: significant after multiple testing).

As Troy is a WNT target gene, we next studied the expression of two other members of the WNT signaling complex, i.e. E-cadherin and β-catenin. Assessment of immunostaining was done as described previously [22]. Thirty-two GCs were classified as E-cadherin- and 50 as β-catenin-positive (Table 1). Interestingly, the expression of Troy in GC correlated highly significantly with the expression of E-cadherin and β-catenin, respectively.

For functional interpretation Troy should be considered a negative regulator of stem cell Wnt signaling. Its expression level might be an indicator for the differentiation commitment of the respective GC cell population. Indeed, Troy expression correlated with the tumor grade (p<0.001) and tumor type (p=0.001) in the entire cohort (104 patients), being significantly more prevalently expressed in well and moderately differentiated GCs and intestinal type GCs (Table 1). Furthermore, expression of Troy correlated significantly with nodal spread (N-category). Node-negative GCs expressed significantly more commonly Troy compared with node-positive GCs (Table 1).

Collectively, these data lead to the conjecture that Troy might also be a prognosticator of patient outcome in that reduced or loss of expression is associated with a worse prognosis. To test this hypothesis, we carried out Kaplan-Meier analyses. These showed a better prognosis for Troy^+^ tumor patients (Figure 3B; p=0.046). As most of these clustered to the intestinal type (IT; Table 1), we further divided the cohort according to their Laurén phenotype. Interestingly, diffuse type GC patients (DT) showed no difference in survival, whereas in the IT group loss of Troy expression was associated with a significantly worse overall survival (Figure 3B; p=0.006). No correlation was found between the expression of Troy in stroma cells (more frequently in IT, Figure 2E and Table 1) and patient survival (Figure 3B). On multivariate survival analysis (Cox regression) four parameters remained in the Cox model after running the backward LR method with p_in_=0.05 and p_out_=0.05. These were patient age, T-category, N-category, tumor grade and resection-status (Supplementary Table S1). Thus, Troy does not prove as independent prognosticator of patient outcome. Other variables, e.g. resection status and nodal spread, outbalance the effect Troy may exert.

Presumably due to the broad expression of Troy in the epithelial and stromal compartment of neoplastic and non-neoplastic gastric tissue, we were unable to establish a predictive qRT-PCR test on *Troy* mRNA levels (Figure 3C). We also followed the hint from a glioma study that correlated *Troy* transcript variant 2 with a poor prognosis albeit a broad Troy expression throughout the adult brain [17]. Nevertheless, repeating the qRT-PCR analysis detecting only this specific variant demonstrated no correlation in GC tissue either (Figure 3C).

Collectively these findings show that Troy is an abundant molecule within the gastric mucosa being co-expressed with other members of the WNT signaling complex and being down-regulated in GC. It is unsuitable as a tumor marker in a simple-to-use manner. However, we propose a careful Troy expression analysis in tumor cells as important determinant for cancer progression and survival prognosis in intestinal type GC.

### Troy expression is positively regulated by Wnt/R-spondin stimulation

**Figure 4:**
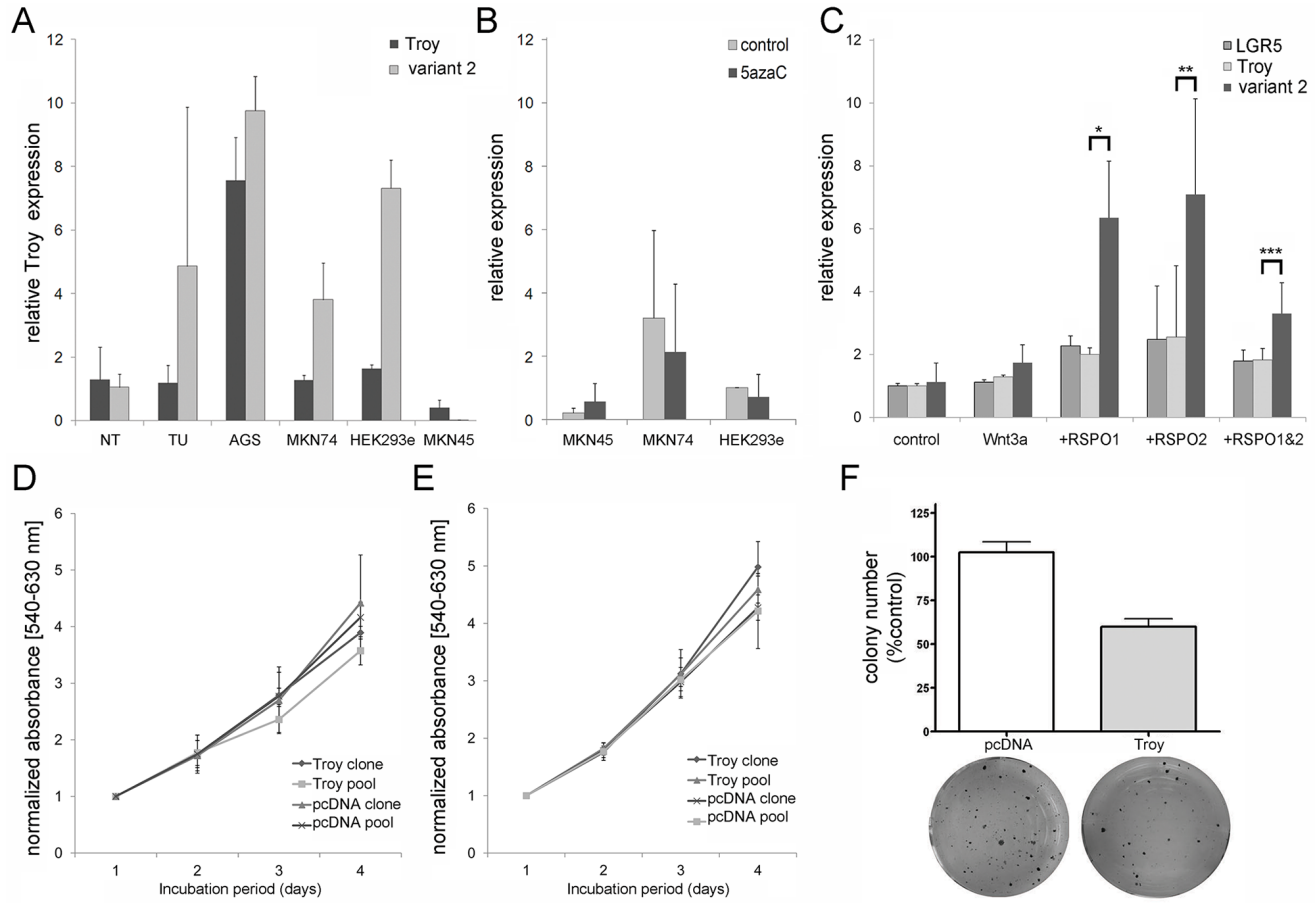
The Wnt target gene *Troy* can be detected in gastric cancer cell lines and overexpression of variant 2 suppresses the tumor phenotype of MKN45 cells **A**. Expression profile of *Troy* and its variant 2 in different cell lines compared with non-neoplastic human stomach tissue [n=3 for non-neoplastic tissue (NT) and tumor (TU), n=2 for cell lines]. Expression was normalized to SDHA in all cell culture experiments. **B**. Expression of Troy in MKN45 cells is not significantly changed after 5azaC treatment for 72 h (n=3). **C**. qRT- PCR analysis of *Troy* and *LGR5* expression after stimulation of MKN45 with 10 nM Wnt3a (n=2) alone or in combination with 10 nM RSPO1 and/or RSPO2 (n=3-4). **D. - E**. MTT based proliferation analysis of stable transfected MKN45 **(D)** and MKN74 **(E)** overexpressing Troy variant 2 compared to control transfected cells (n=3). **F**. Colony formation assay evaluates the ability for clonal expansion of Troy variant 2 transfected MKN45 (n=8). All data are represented at mean +/- SD with *p=0.051; **p=0.108; ***p=0.031).

Next we carried out cell culture experiments in order to explore putative tumor cell biological functions of Troy. In agreement with the finding of Troy in GC, a variable amount of Troy expression was found in three GC cell lines (AGS, MKN45, MKN74) as well as in HEK293ebna cells (Figure 4A). Especially transcript variant 2 showed elevated levels in MKN74 and AGS cells, whereas MKN45 cells resembled the overall Troy expression of healthy stomach tissue with low expression of *Troy* transcript variant 2. To examine whether *Troy* expression was modulated by epigenetic mechanisms, demethylation was induced by 5azaC treatment in three different cell lines. Evaluation of the transcriptional activity by qRT-PCR on *Troy* mRNA showed no differences between treated and control cells (Figure 4B) indicating that *Troy* expression is controlled by other mechanisms than methylation. We furthermore validated *Troy* as a target gene of an active Wnt signaling by stimulating MKN45 with a cocktail of Wnt3a and R-spondins 1 and 2 (RSPO 1+2; Figure 4C). Interestingly, both RSPOs induced *Troy* expression to a comparable extent, favoring *Troy* variant 2 transcription over other Wnt target genes like LGR5.

### Troy may act as negative regulator of tumor-promoting functions

**Figure 5:**
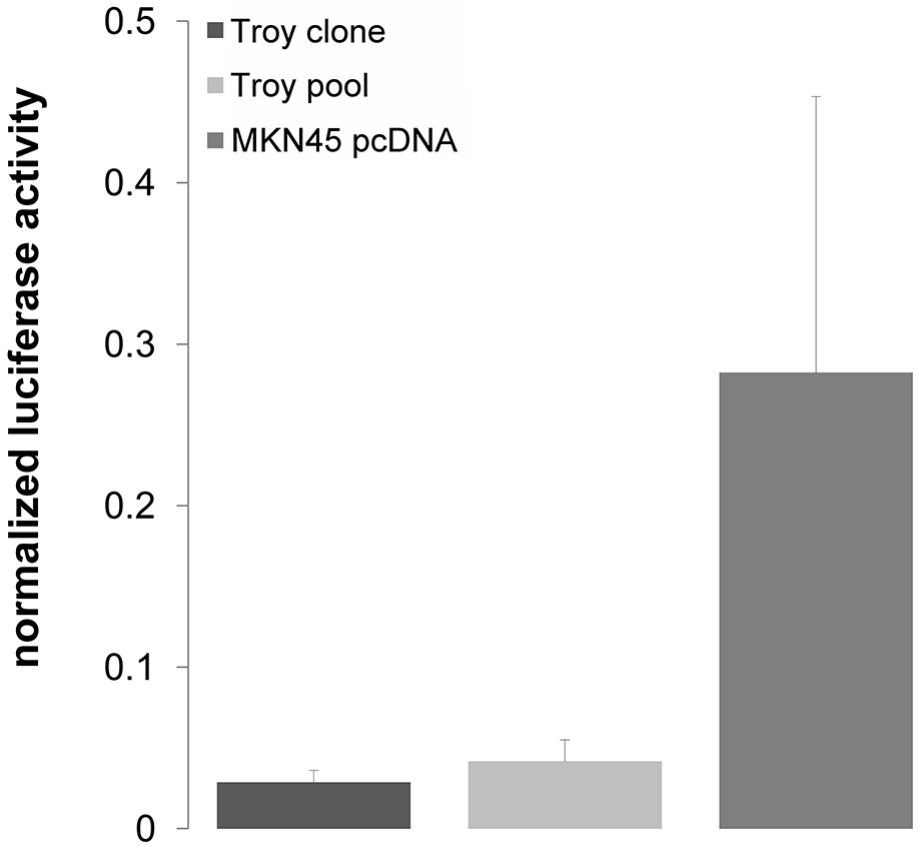
Troy variant 2 suppresses Wnt target gene expression After 20 h of stimulation with 10 mM LiCl, TCF/Lef1 dependent reporter activity measured by luciferase signal is decreased in Troy overexpressing MKN45-cells compared with the pcDNA control line (n=2 independent experiments; mean ± SD; p=0.001).

Given that especially *Troy* transcript variant 2 shows strong induction by Wnt signaling and associations to cancer progression, MKN45 and MKN74 cells were used to generate stable cell lines with *Troy* variant 2 overexpression (Supplementary Figure S1). Troy overexpressing MKN45 and MKN74 cells showed no significant difference in cell proliferation compared with control cells (Figure 4D & 4E). However, the ability for clonal expansion was reduced in Troy transfected MKN45 cells (Figure 4F) and unaffected in Troy-transfected MKN74 cells, identifying Troy as a putative negative regulator of stem cell properties in MKN45. In a subsequent TCF/Lef1 reporter assay, we could prove Troy to reduce β-catenin dependent gene transcription (Figure 5), thereby silencing Wnt signaling.

Taken together, we showed for the first time that Troy protein is abundantly expressed in differentiated cells of the human gastric mucosa. As Troy is a negative regulator of Wnt signaling [9] and thereby modulates stem cell activity, reduced expression in GC might indicate loss of cell differentiation, thereby affecting patient prognosis.

## DISCUSSION

The Wnt signaling pathway is a critical regulator of stem cell function in gastrointestinal tissues and is controlled via multiple regulatory ligands [23, 24]. The resulting level of free β-catenin dictates the degree of gene expression and first evidence suggests that the final signal strength is translated into an on/off signal of a special subset of transcription factors controlling stem cell identity and differentiation fate [25]. One relevant Wnt target gene is Troy, a transmembrane protein of the TNF receptor superfamily [11]. Interestingly, this protein exhibits a dual function by activating NFkB signaling [20] or negatively influencing the Wnt signaling cascade [9], possibly depending on the cellular context.

Here, we show for the first time that Troy is abundantly expressed in the human adult stomach on transcriptional and protein level. We identified differentiated parietal and chief cells as main population of Troy^+^ cells in the healthy human corpus gland. This resembles the expression profile of the TROY-CreERT2 mouse model, which correlated a subset of Troy^+^ chief cells with the capability to regenerate the whole stomach epithelium [21]. In the human stomach, we found Troy^+^ cells to even greater extent, what might in parts be explained by a mosaic expression pattern and further translational regulation, as these mice only show the transcriptional activity of the promoter, not the presence of the protein.

Furthermore, *Troy* gene expression might be regulated in a more complex manner in humans compared to mice. Originally identified as a TCF4-dependent Wnt target gene [9, 26], *Troy* expression seems to be additionally or alternatively controlled. In the human stomach we could detect Troy in differentiated parietal and chief cells and in a significant proportion of GC cells. We demonstrated the ability of Wnt3a/RSPOs to elevate *Troy* mRNA levels in gastric cancer cell lines and found no evidence for an epigenetic modulation. Interestingly, transcript variants 1 and 2 differentially responded to Wnt pathway stimulation. This is comparable to a finding in colorectal cancer, where both transcript levels increased upon incubation with Wnt ligand, but mutation of the LEF/TCF motifs prevented only variant 2 (but not variant 1) promoter activity [20]. Further research will be needed to identify the relevant transcriptional regulators apart from the Wnt/β-catenin pathway leading to such a distinct expression profile of *Troy*.

The resulting Troy protein variants 1 and 2 show high similarity and differ only in their very C-terminal amino acid composition with the shorter variant 2 consisting a TRAF2-binding sequence that is missing in variant 1. Via this binding domain, Troy is able to activate NFkB signaling [11, 14], however in colorectal cancer cell lines both variants were able to induce NFkB reporter activity [20] arguing for further signaling modes. This view is even more challenged, as in glioma patients especially transcript variant 2 has been associated to Rac1 signaling stimulating migration and leading to a poorer outcome [17, 27]. In our GC cohort, we were unable to detect differences in the expression levels of both *Troy* variants although a first random test showed a trend to a higher *Troy* variant 2 level (Figure 4A). We hypothesize that the analysis is hampered by the broad expression of *Troy* within the gastric mucosa as well as muscle and endothelial cells, with competing expression trends within the cell populations.

Nevertheless, analyzing the spatial distribution of Troy by immunohistochemistry we found a distinct Troy expression in the tumor cell population as well as in the neoplastic stroma (Table 1). Both strongly correlated with the tumor grade and histological phenotype of GC. Notably, loss of Troy expression in tumor cells was associated with poor patient outcome. Troy might therefore be used as determinant of cellular commitment, which is in good agreement with the finding of intestinal metaplasia often expressing high levels of Troy (Figure 2F). In addition, our results lend support to the contention that Troy expression leads to functional changes of GC cell biology.

Although the exact mechanism is hardly understood, Troy is suggested to inhibit the Wnt signal by decreasing LRP phosphorylation [9], and to adjust the Wnt signal strength to enable the maintenance of stem cell functions as well as tissue homeostasis [28]. Knockdown of Troy shows no obvious severe phenotype and a normal gut development [14] implying a moderate regulatory function. However, we could prove that overexpression of Troy in the GC cell line MKN45, which harbors the non-mutated wild type *apc* and *ß-catenin* genes [29], led to decreased clonal expansion ability. Clonal expansion was not significantly affected in Troy-overexpressing MKN74-cells, which are known to carry an *apc* mutation, further illustrating that Troy may only have a modulating effect on WNT signaling. However, these observations emphasize the role of Troy as a negative regulator of Wnt driven stem cell functions and led us to hypothesize a role of an increased Troy expression in shifting cellular processes towards a differentiation fate in the stomach tissue. Only recently, the significance of a Runx2-dependent Troy expression for osteoblastogenesis was shown [30] and in human mesenchymal stem cells variant 2 was correlated with osteoblast differentiation, whereas Troy variant 1 was shown to inhibit adipocytic differentiation [31]. Therefore, a complex mechanism might underlie the expression of Troy in humans resulting in a context dependent intracellular signaling, a decrease in Wnt activity and finally the initiation of differentiation.

Interestingly, we were unable to observe an effect of Troy on cell proliferation. This result is in line with previous findings of our group [32]. There was no significant correlation between the Ki67-proliferation index and overall or tumor specific patient survival. Thus, cell proliferation *per se* is not as important for GC biology as it is for various other types of cancer [32].

In summary, we provided data for an unexpected broad expression of Troy in differentiated cell types of the human stomach tissue. Using *in vitro* experiments, we showed a Wnt/RSPO dependent expression of *Troy* and confirmed its negative function on stem cell properties. As we already established LGR5 as a predictive marker of putative cancer stem cells within the gastric mucosa [33], we now show that the expression of its antagonist Troy might improve the patient's outcome by inducing differentiation. Its precise molecular regulation in the particular cellular context remains to be elucidated.

## MATERIALS AND METHODS

### Study population and ethical statement

Non-neoplastic and neoplastic gastric tissue was obtained as part of a therapeutic surgery. Patient identifiers were kept confidential and analyzed with the approval of the local ethics committee of the University Hospital Kiel, Germany (reference number D 452/11 and 472/15). The study cohort retrieved from the institute's archive material (years 1997 to 2014, patient characteristics summarized in Table 1) contained 104 paraffin-embedded samples (52 of them with paired normal tissue control), 50 paired unfixed fresh frozen tissue sections for qRT-PCR analysis as well as 5 sleeve gastrectomy specimens without neoplastic transformation to study the naïve expression pattern of Troy. The entity of randomly picked cases was controlled for a representative balance of TNM staging compared to our institute's European stomach cohort. Each GC specimen was histologically examined by a trained surgical pathologist and classified according to the Laurén classification [34] and the 7^th^ edition of the union internationale contre le cancer (UICC) [35]. Date of patient death was obtained from the Epidemiological Cancer Registry of the state Schleswig-Holstein, Germany.

### Immunohistochemistry and staining evaluation

Formalin-fixed and paraffin-embedded (FFPE) tissue sections were pretreated in citrate buffer for antigen retrieval and incubated with hydrogen peroxide block and Ultra V Block Staining (both Thermo Scientific, Braunschweig, Germany) to avoid unspecific reactions. Troy immunohistochemistry was performed using a monoclonal anti-TROY antibody (dilution 1:1600, ab138502, Abcam Inc., Cambridge, USA). Antibody specificity was verified with an already FFPE-established antibody (1:50, HPA010135, Sigma-Aldrich, Munich, Germany) at serial sections. For double staining, the following antibodies were used: H^+^/K^+^-ATPase (1:2000, 2G11, Thermo Scientific), pepsinogen C (1:100, ABIN1999516, antibodies-online, Aachen, Germany), Muc5AC (1:100), Muc6 (1:40), chromogranin A (1:100, all Leica Biosystems, Nussloch, Germany). For visualization, anti-rabbit-HRP, anti-mouse-AP-polymer (both Abcam, Cambridge, UK) or ImmPRESS-universal-HRP-polymer combined with the NovaRED, VectorRED or DAB substrate kit (all VectorLabs, Peterborough, UK) were applied. To evaluate number and intensity of Troy^+^ cells within the gastric mucosa, the Histoscore system [36] was applied: briefly, immunoreactive cells were surveyed for 4 categories documenting the intensities of the immunostaining, i.e. 0 (negative), 1 (weak), 2 (moderate) and 3 (strong), and the percentage of immunostainend cells. Using the formula [%]x0+[%]x1+[%]x2+[%]x3 the total score with values between 0 and 300 was calculated. Staining of immune and stromal cells or myocytes was recorded separately. To divide groups of high and low/negative expression, Histoscore results were splitted at the median.

Immunostaining and evaluation of E-cadherin (clone SPM471; ZYTOMED Systems GmbH, Berlin, Germany; 1:400) and β-catenin (clone Cat-5H10; Life Technologies GmbH, Darmstadt, Germany; 1:300) was done as described previously [22].

### Immunocytochemistry

Stable transformed cell lines were harvested and centrifuged for 5 min at 1200 rpm, the resulting pellets were washed in PBS and resuspended in 10 % PBS buffered formalin. After 24 h formalin fixation, cells were centrifuged for 5 min at 1200 rpm. The supernatants were discarded and the cells were pre-embedded in low-melting agarose (Sigma-Aldrich). The resulting agarose-drops were placed in a tissue cassette and subjected to routine paraffin embedding process using the Hypercenter Tissue Processor (Shandon, Ramsey, USA). TROY immuncytochemistry was done as described using the anti-TROY antibody (Abcam, ab137080, dilution 1∶500).

### Reverse transcriptase reaction and quantitative real-time PCR (qRT-PCR)

Total cellular RNA from cell culture samples was isolated using the High Pure RNA Isolation Kit from Roche (Mannheim, Germany). RNA from gastric tissues was prepared with the mirVana Isolation Kit followed by DNase treatment with the Turbo DNA-free kit (both from LifeTechnologies, Darmstadt, Germany). RNA concentration was measured using a NanoDrop 2000 device (Thermo Scientific, Darmstadt), integrity was assessed by agarose gel electrophoresis and purity was controlled by PCR on the genomic sequence of ß-actin. Reverse transcription of 1 μg RNA was carried out with Maxima First Strand cDNA Synthesis Kit (LifeTechnologies) according to the manufacturer's instructions. For qRT-PCR analysis of cell culture experiments the QuantiTect SYBR Green PCR Kit (Qiagen, Hilden, Germany) was applied on a 1.5 LightCycler System (Roche). Tissue analysis was performed with the LightCycler 480 Probes Master (Roche, housekeeping genes) or the Maxima Probe qPCR Master Mix (LifeTechnologies, *Troy* variants) and the according probe and primer sets (Universal Probe Library, Roche, cat. no. 04683633001, see Table 2) on a LightCycler 480 Instrument II (Roche).

**Table 2:** Primer sequences. For qRT-PCR detection the roche universal probe library was applied

Target	Forward sequence (5’ => 3’)	Reverse sequence (5’ => 3’)	Probe	Application
Troy, variant 2	gagagctaagcatgggtttaaaagtgctactagaacaaga	gagactcgagttaagcttcctggagggacgtct		cloning
β-actin	gccatgtacgttgctatcca	ctccttaatgtcacgcacga		gDNA
Troy (all variants)	ggagtgtgtgccttgtgga	gcgatcttcacgaggttga	#63	qRT-PCR
Troy (variant 2)	tagaagcatttggcacagaagt	caaatgtatttattgttggagagttcc	#2	qRT-PCR
SDHA	atttggtggacagagcctca	ctggtatcatatcgcagagacc	#5	qRT-PCR
CYCC	ggaaaagtcattgatgggatg	caaaaggcgttttcacgtcta	#59	qRT-PCR
CAPN2	cgctgacccccagtttatc	tcaaggtgagggaggcaat	#25	qRT-PCR

### Cloning of the troy expression construct

The open reading frame of human TROY.2 (NM_148957) was amplified from MKN45 cDNA (see Table 2 for primer sequences), introduced to pcDNA3.1(-) vector using the NheI and XhoI restriction sites and controlled for correct integration by sequencing with the ABI PRISM BigDye Terminator Cycle Sequencing Ready Reaction Kit (PE Applied Biosystems, Langen, Germany).

### Cell culture procedures and generation of stable transformants

The human gastric cell lines MKN45 (German Collection of Microorganisms and Cell Cultures, DSMZ, Braunschweig, Germany) and MKN74 (Japanese Health Science Research Resource Bank, Osaka, Japan) were cultured in RPMI supplemented with 20% or 10% fetal calf serum (FCS GOLD; PAA Laboratories, Pasching, Austria). HEK293ebna (Invitrogen, Carlsbad, USA) were maintained in Dulbecco's modified Eagles Medium (DMEM, 10% FCS). For generation of stable Troy expressing cell lines, MKN45 and MKN74 were transfected using Lipofectamine LTX (Invitrogen) and cultured under selection pressure with 650 μg/ml (MKN45) or 450 μg/ ml (MKN74) G418 (PAA) as described previously [33]. Overexpression was verified by qRT-PCR, immuncytochemistry and Western blotting (Supplementary Figure 1). For MKN45 Wnt signaling was induced at day 0 and 2 by incubating cell cultures with the recombinant proteins Wnt3a (BioCat, Heidelberg, Germany), RSPO1 (LifeTechnologies) or RSPO2 (antibodies-online) and analyzed at day 4. For demethylation studies, subconfluent cell cultures were daily treated with 1 μM 5-Aza-2’-Deoxycytidine (5azaC; BioCat) and expression profile was analyzed after 72 h.

### Proliferation and colony forming assay

Proliferation rates were studied with a 3-(4,5-dimethylthiazolyl)-2,5-diphenyltetrazolium bromide (MTT) based assay. Briefly, 5×10^3^ cells were seeded into 96 well plates and analyzed with fresh MTT solution (0.5 mg/ml; Sigma-Aldrich, Taufkirchen, Germany) after 24, 48, 72 and 96 h. Following centrifugation (10 min 1500 rpm), supernatant was removed and crystals solubilized in 100 μl dimethylsulfoxide. Absorption measurement was performed at the Synergy Mx device (Biotek, Bad Friedrichshall, Germany) at 540 nm and 680 nm for reference.

The colony forming assay was performed according to an established protocol [37]. MKN45 and MKN74 cells were seeded onto 6-well dishes and transfected with the Troy expression vector or empty pcDNA3.1(-). G418-resistant clones were selected over a period of 17 days as described above. For analysis, clones were fixed with 4% formalin and stained with 0.1% crystal violet. After thoroughly rinsing, colony number was determined via the “ColonyCount” function at the XR Gel Station (BioRAD, Munich, Germany) with sensitivity 6,7 and average 7, to assess only clearly visible clones (assumably more than 50 cells).

### TCF/Lef1 luciferase reporter gene assay

Reverse transfection was performed with stable MKN45 transformants by using Lipofectamine LTX (Life Technologies) according to the manufactures high-throughput Protocol. 4×10^5^ Cells were seeded in 96 well plates and transfected using 100 ng of the Cignal TCF/LEF Reporter (Qiagen). 24 h after transfection, cells were treated with 10 mM lithium chloride (LiCl; Merck Chemicals, Darmstadt, Germany) for 20 h. Firefly luciferase reporter activity was measured using the Dual Glo luciferase assay System (Promega, Mannheim, Germany) and normalized to Renilla luciferase activity.

### Western blot

Protein lysates were obtained by lysis of cultured cells in RIPA buffer (Sigma-Aldrich) including protease inhibitor cocktail (Complete EDTA-free, Roche). Protein samples were denaturated in Laemmli buffer (60 mM Tris-HCl pH 6.8, 2% sodium dodecyl sulphate, 10% glycerol, 5% β-mercaptoethanol and 0.01% bromphenol blue) by heating at 95°C for 10 min and were subsequently loaded on 4%-15% Mini-PROTEAN TGX Precast Gels (BioRAD). After separation, proteins were transferred to a nitrocellulose membrane (Amersham, Freiburg, Germany) and immunoblotted with the anti-TROY-antibody (dilution 1:500; ab137080; Abcam) and an anti-β-actin-antibody (1∶30,000; clone AC-15; Sigma-Aldrich) to ensure equal loading amounts. Membrane bound HRP labelled secondary antibodies (DakoCytomation, Glostrup, Denmark) were detected by enhanced chemiluminescence using the ECL system (Amersham).

### Statistical analyses

Statistical analyses were done using SPSS 20.0 (IBM Corporation, New York, USA) and GraphPad Prism4 (GraphPad Software, Inc., La Jolla, USA). For comparison purposes, the Troy-Histoscore evaluated by WTS was dichotomized at the median; patients above median were classified as “Troy-positive”, patients below median were classified as “Troy-negative”. For histochemical analysis, grouped data was analyzed using the 2-sided Fisher's exact test for nominal variables and Kendall's tau rank-order correlation for ordinal variables. A p-value ≤ 0.05 was considered statistically significant. Effects of multiple testing were accounted for by applying the explorative Simes (Benjamini-Hochberg) procedure [38]. All p-values are given unadjusted but are marked where they lose significance under the Simes procedure. The median overall survival was determined by the Kaplan-Meier method and significance was assessed by the log rank test. For cell culture data, the Mann-Whitney U-Test or Student's T-test was applied.

## SUPPLEMENTARY MATERIALS FIGURE AND TABLE


