# Supplementary material for: Troy is expressed in human stomach mucosa and a novel putative prognostic marker of intestinal type gastric cancer
Source: Oncotarget. 2016 Jul 18;8(31):50557–69. doi: 10.18632/oncotarget.10672 (PMC5584167; doi:10.18632/oncotarget.10672)
Supplement: Supplementary file 1 [file oncotarget-08-50557-s001.pdf]

Troy is expressed in human stomach mucosa and a novel putative prognostic marker of intestinal type gastric cancer

SUPPLEMENTARY FIGURE AND TABLE

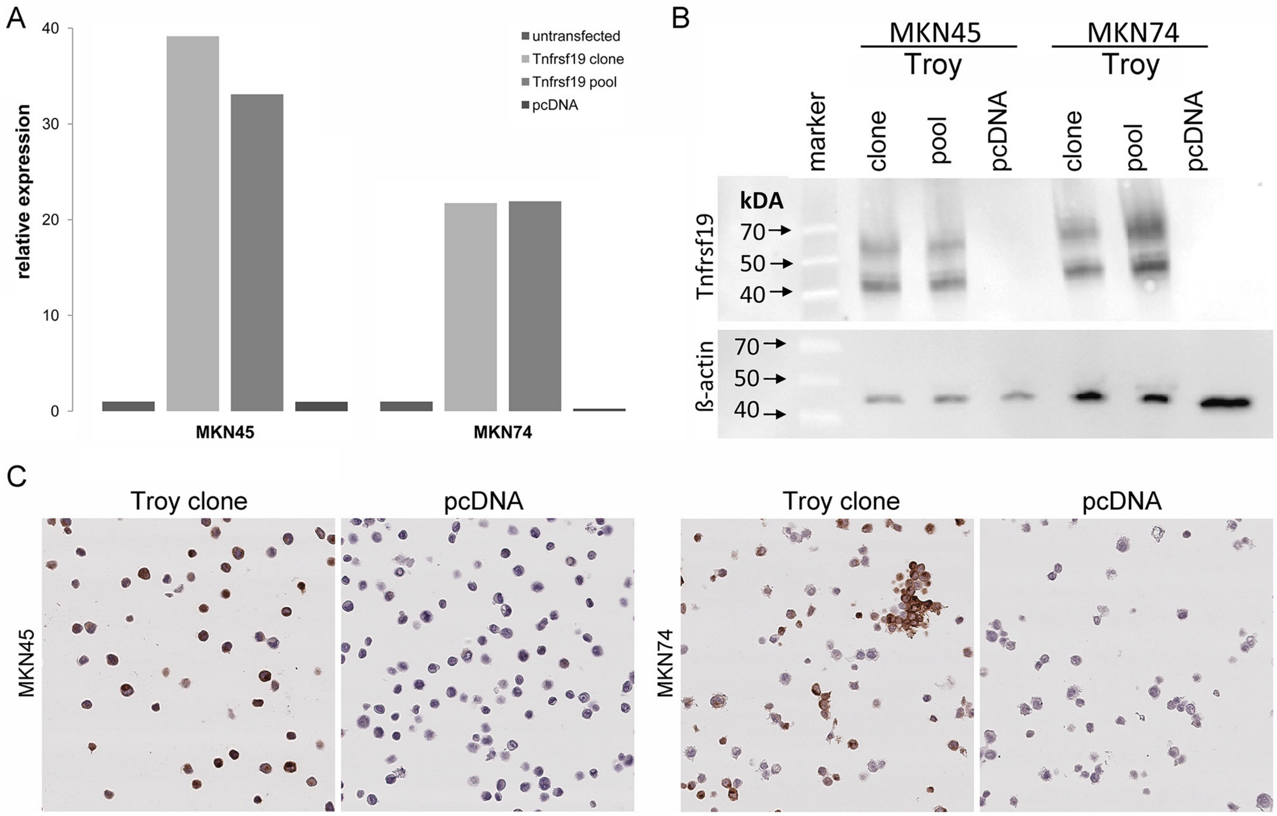

Supplemental Figure 1: Stable transfection of MKN45 and MKN74 cells with Troy variant 2. A. MKN45 and MKN74-cells were stable transfected with Troy and verified by qRT-PCR. **B.** Troy overexpression detected by Western blotting **C.** Immunocytochemistry using an anti-Troy-antibody in agarose embedded transformants (original magnifications 400x). "Pool" denotes all cells, which had incorporated the plasmid. "Clone" denotes a single cell clone which incorporated the plasmid.

Supplementary Table S1: Multivariate survival analysis

	Overall survival *			Tumor specific survival *		
	**HR**	**95% C.I.**	**p-Value**	**HR**	**95% C.I.**	**p-Value**
Age ≤68 years vs. > 68 years	2.580	1.563-4.257	<0.001			
T-category						
T2 vs. T1	3.346	0.394-28.391	0.268			
T3 vs. T2	1.861	0.762-4.543	0.173			
T4 vs. T3	2.160	1.261-3.701	0.005			
N-category			0.010			0.022
N1 vs. N0	1.901	0.951-3.800	0.069	2.045	0.895-4.672	0.090
N2 vs. N1	0.579	0.242-1.383	0.219	0.540	0.210-1.390	0.201
N3 vs. N2	2.448	1.110-5.397	0.026	2.385	1.048-5.431	0.038
G3/G4 vs. G1/G2	2.665	1.548-4.588	<0.001			
R1/R2 vs. R0	11.729	4.427-31.075	<0.001	10.124	3.819-26.833	<0.001

*Input variables: Parameters which had a p<0.05 in univariate survival analysis were patient age, Laurén-phenotype, T-, N-, M-, and R-category, UICC-stage, tumor grade, and Troy-expression in tumor cells.
